# Bernese Periacetabular Osteotomy: A Comparative Study of Four Types of Incisions

**DOI:** 10.5435/JAAOSGlobal-D-21-00090

**Published:** 2022-12-05

**Authors:** Joaquín Lara, Alan Garín, Cristhián Herrera, Selim Abara, Jaime Cancino, Hassan Neumann, Javier del Río, Javier Besomi, Diego Villegas, Carlos Tobar

**Affiliations:** From Clínica MEDS (Dr. Lara and Dr. del Río), Instituto Traumatológico (Dr. Garín), Hospital Clínico Mutual de Seguridad CChC (Dr. Cancino and Dr. del Río), Hospital Padre Hurtado (Dr. Neumann and Dr. Villegas), Clínica Alemana de Santiago (Dr. Herrera, Dr. Neumann and Dr. Besomi), Clínica RedSalud Providencia (Dr. Tobar and Dr. Villegas), Hospital Clínico San Borja Arriarán (Dr. Herrera and Dr. Besomi), Clínica RedSalud Santiago (Dr. Garín), Santiago, Chile; and Hospital DIPRECA, Las Condes, Chile (Dr. Abara).

## Abstract

Bernese periacetabular osteotomy has diverse complications associated with incisions, such as dehiscence, hypertrophy, depression, and hyperpigmentation on scars, which affect patient satisfaction. The objective was to evaluate aesthetics and satisfaction outcomes in four different incisions. We evaluated 176 incisions in 148 patients. The incisions performed were the original modified (16, group I), straight and shortened, (64, group II), “Z” shaped (16, group III), and oblique inguinal (48, group IV). The scars were evaluated for width and length, development of a hypertrophic scar, depression or hyperpigmentation, and dehiscence and resuture. A scale of satisfaction was applied (points ranging from 1 to 10). The Bartlett test and Kruskal-Wallis test were used. The mean width and length of the scars were 20.3 and 6.8 cm for group I, 6.5 and 8.1 for group II, 12.1 and 7.1 cm for group III, 13 and 1.4 cm for group IV, respectively. Hypertrophic scars were found in 18% in group I, 12.5% in group II, and 31.2% in group III. Depressed scars were found in 10.8% in group I and 7.1% in group II. Hyperpigmentation was found in 16% in groups I and II, 37% in group III, and 2% in group IV. Dehiscence was found in 8.1% in group I and 8.9% in group II. Satisfaction for group IV was nine points. The difference in length and width and satisfaction were statistically significant (*P* < 0.05). The oblique inguinal incision (group IV) showed a smaller percentage of complications, with an adequate aesthetic result, and a high grade of patient's satisfaction.

Among the complications reported for Bernese periacetabular osteotomy are those related to the surgical wound and healing process. In the publication on the anterior hip and pelvic approach for osteotomies and acetabular and femoral injuries, Ganz and Weber^1^ described the formation of a large scar distal to the anterosuperior iliac spine (ASIS) as a disadvantage of this approach and highlighted the importance that this has for young patients. These scars can be wide, hypertrophic, and notorious. These aesthetic problems are added to events of wound dehiscence that may even require reinterventions.

The surgical technique has been modified to avoid these complications, particularly in relation to the original Smith-Petersen approach.^2^ In the original technique, this approach is conducted by making a longitudinal incision along the iliac crest, passing through the ASIS, and going toward the lateral thigh region, which is approximately 20 to 25 cm long (Figure 1). Various modifications^3^ have been made to this approach, such as direct anterior, shortened rectus, ilioinguinal,^4^ transartorius,^5^ minimally invasive,^6^ and double approaches,^7^ among others, but these have not fully resolved the complications arising from the surgical wound and the appearance of the scar, which, in our experience, translates into both functional and patient satisfaction problems.

**Figure 1:**
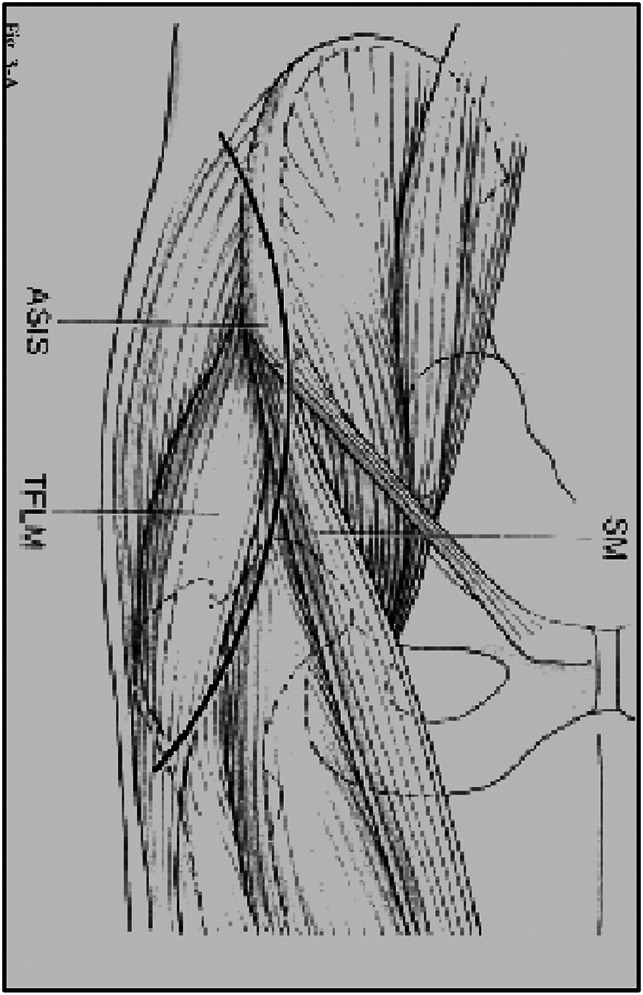
Diagram showing a longitudinal incision.

To avoid these complications, our group has carried out different types of incisions for the anterior hip approach in periacetabular osteotomy. From 2006 to 2017, the main author made four different types of incisions, starting with the classic longitudinal incision described earlier, followed by the shortened straight incision, which extends from the ASIS for 6 to 8 cm distally (Figure 2); the “Z” incision (Figure 3); and finally the oblique inguinal incision^8^ (Figure 4). The straight and shortened incision (group II) and Z-shaped incision (group III), as most of the modified approaches to perform this technique, were developed with the objective of obtaining shorter wounds and scars. However, the longitudinal orientation of these incisions, perpendicular to the skin tension lines described by Langer^9^ and Kraissl^10,11^ for the inguinal region (Figure 5), resulted in wounds that, similar to the original technique, maintained their tendency to separate the edges and frequently generate wide scars with trophism defects. Considering these characteristics of the skin in the area of the surgical approach, the oblique inguinal incision was developed, whose technique and results were presented in a previous study.^8^ The main characteristic of this incision is that it is oriented in the direction of the skin tension lines described by Langer and Kraissl, decreasing the static and dynamic tension of the skin, which allows the edges of the wound to tend to spontaneous closure and, therefore, avoids the development of dehiscent wounds or aesthetically unsatisfactory scars. In addition, because of its location, the scar is easily hidden under underwear, a situation highly valued by patients.

**Figure 2:**
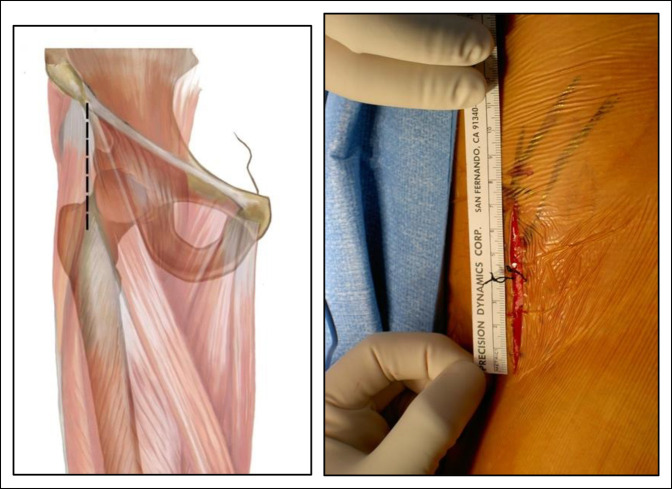
Illustration and photograph showing a shortened straight incision. It extends from the ASIS 6 to 8 cm distally. ASIS = anterosuperior iliac spine

**Figure 3:**
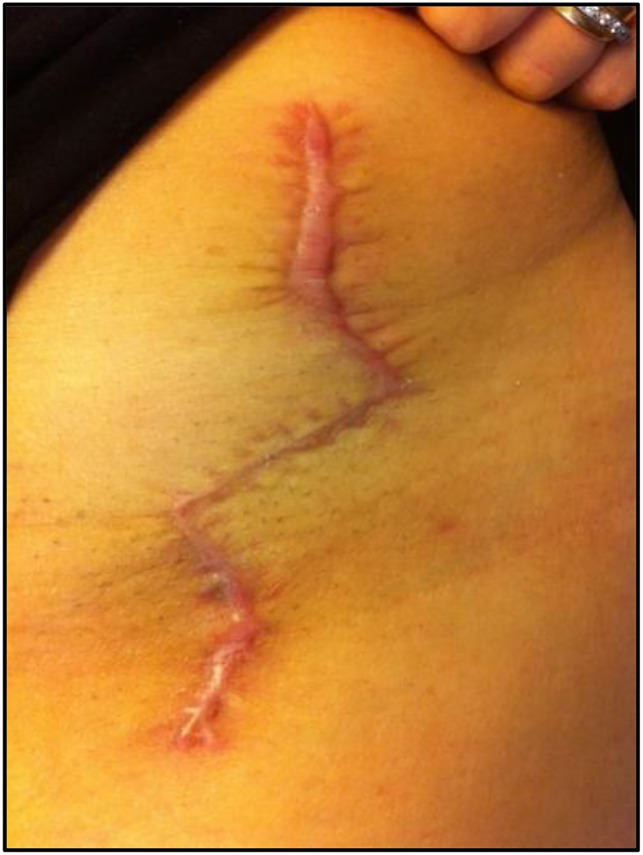
Photograph showing a Z incision.

**Figure 4:**
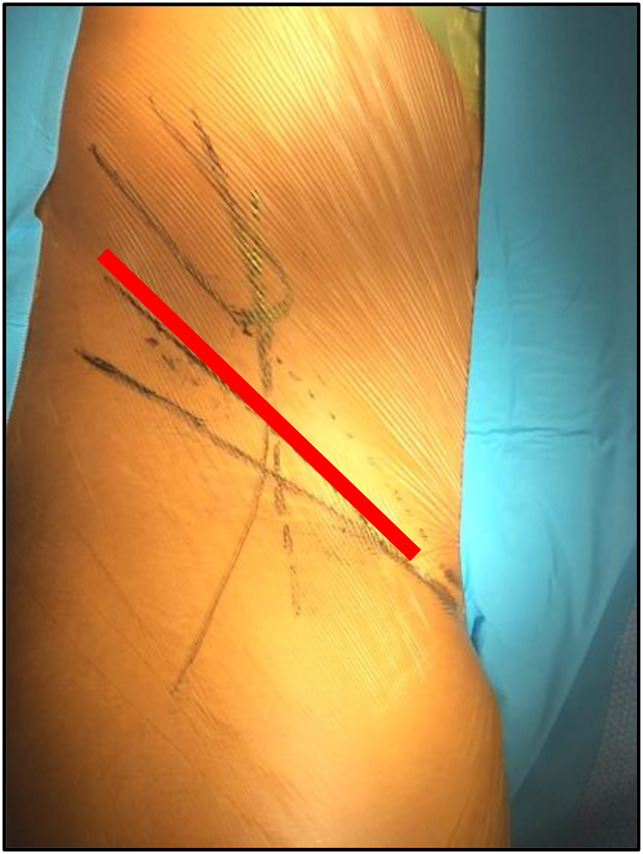
Photograph showing an oblique inguinal incision.

**Figure 5:**
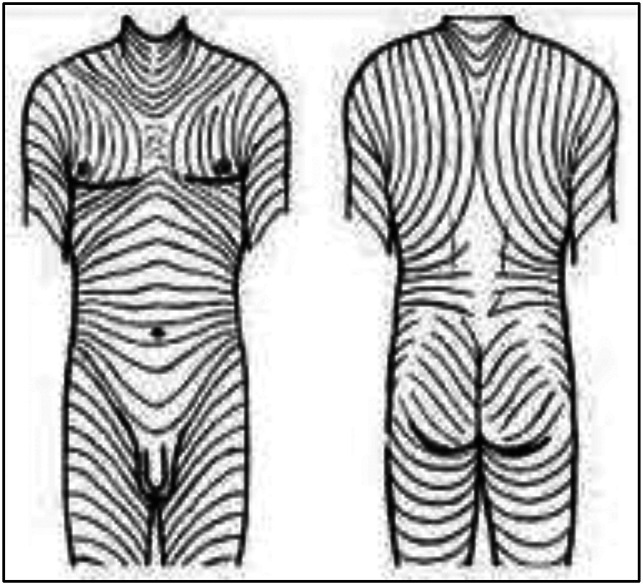
Illustration showing Langer lines.

## Objectives

The objective of this study was to compare the four types of incisions for the modified anterior approach with the Ganz periacetabular osteotomy, evaluating scar characteristics, associated complications, and patient satisfaction.

## Methods

This is a descriptive study. Between July 2006 and May 2017, the main author conducted 195 periacetabular osteotomies in 167 patients. The group included 173 periacetabular osteotomies in 148 consecutive patients. Nineteen patients (22 osteotomies) who could not be contacted or did not attend the medical evaluation control were excluded. In all of them, a Smith-Petersen approach was conducted, with the technique modified by the main author.^12^ One hundred thirty female patients and 18 male patients were included in this study, with the age range of 14 to 46 years (average 26.5 years). Forty-seven longitudinal incisions were performed in 40 patients (34 women, 6 men, mean age 28 years, mean body mass index [BMI] 23.47), 64 shortened straight incisions in 54 patients (50 women, 4 men, mean age 26.07 years, mean BMI 23.97), 14 Z-shaped incisions in 14 patients (12 women, 2 men, mean age 27.3 years, mean BMI 24.1), and 48 oblique incisions in 40 patients (34 women, 6 men, mean age 25.3 years, mean BMI 24.26). The minimum follow-up was 6 months for all patients (range 6 to 141 months).

The characteristics of the scar were evaluated in postoperative control subjects with a minimum of 6 months after surgery. The characteristics such as measurement of the length and diastasis of the scar edges were evaluated (recording the average between the widest and narrowest parts of the scar). The frequency of the development of hypertrophic, depressed, and hyperpigmented scars was qualitatively evaluated. In addition, the events of dehiscence and resuture were recorded. A patient satisfaction survey was conducted in relation to their aesthetic opinion of the scar by means of a subjective numerical scale (0 = very dissatisfied, 10 = very satisfied).

The data obtained were tabulated in Microsoft Excel software, and Stata/IC v.15.1 for Mac was used for obtaining descriptive statistics for each covariate and testing for statistical significance. The statistical tests used were analysis of varianceby the Bartlett's test and the Kruskal-Wallis test.

## Results

**Figure 6:**
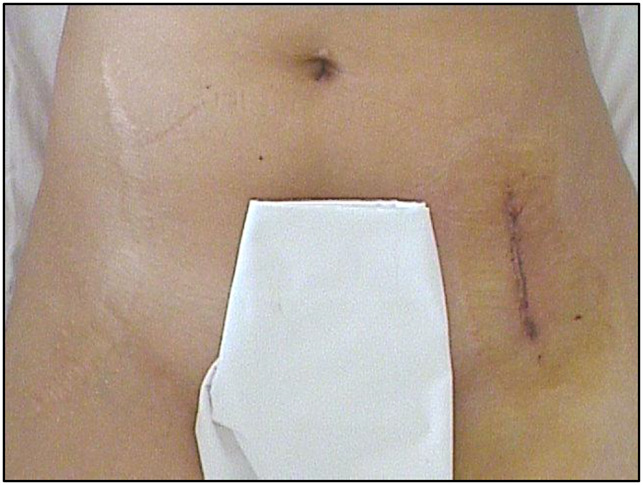
Image showing a longitudinal incision scar.

**Figure 7:**
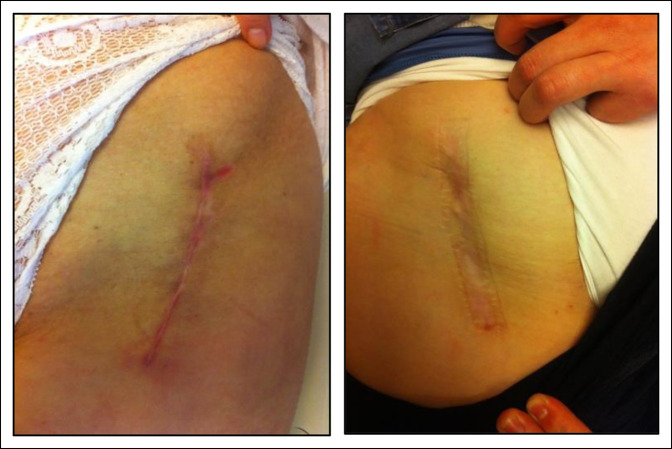
Images showing a shortened straight incision scar.

**Figure 8:**
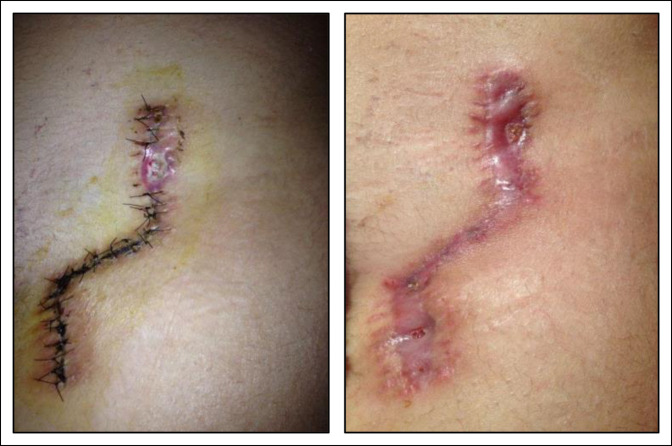
Images showing a Z-shaped incision scar.

**Figure 9:**
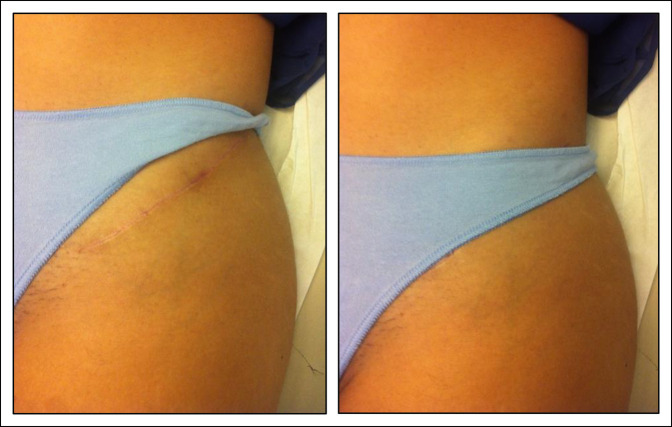
Images showing an oblique incision scar.

Forty-seven osteotomies were conducted with the longitudinal incision (group I) (Figure 6), 64 with the shortened straight incision (group II) (Figure 7), 14 with the Z-shaped incision (group III) (Figure 8), and 48 with the oblique incision (group IV) (Figure 9). The results of the characteristics evaluated by each group are summarized in Table 1. Notably, the oblique incision presents significantly less scar width and greater aesthetic satisfaction of the patients when compared with the rest of the incisions made.

**Table 1:** Summary of Results

	Group I (n: 47)	Group II (n: 64)	Group III (n: 14)	Group IV (n: 48)
Length (cm)	20.3	6.5	12.1	13.1
Width (mm)	6.8	8.1	7.1	1.4^a^
Hypertrophy	18%	12.5%	31.2%	0%^a^
Depression	10.8%	7.1	0	0^a^
Hyperpigmentation	16%	16%	37%	2%^a^
Dehiscence	8.1%	8.9%	12.5%	0^a^
Satisfaction	6	5	5	9^a^

a Statistically significant.

## Discussion

Among the complications of the Ganz periacetabular osteotomy are those related to the surgical wound and healing process. The patients who undergo this surgery are mostly young female patients, so the aesthetic results become very important. The evolution of dehiscent wounds and large scars are problems that both the surgeon and the patient must face.

In the original technique described by Ganz, an anterior hip approach is conducted through a longitudinal incision of approximately 20 to 25 cm, which allows wide exposure and visualization for the performance of osteotomies. However, this very long incision, perpendicular to the skin tension lines described by both Langer and Kraissl,^11^ generates wounds with edges that naturally tend to separate, thus resulting in large scars (in width and length) and poor aesthetics. In addition, given the notable injury to the soft parts in the classic anterior approach, the functional result and therefore the rehabilitation of these patients may be delayed when compared with other approaches.^13^

The modifications of the approach and the incisions for the Ganz osteotomy have had as a main objective to obtain scars of shorter length and better aesthetic results. This is the reason for the development of the shortened straight and Z-shaped incisions in our group. Despite fulfilling the objective of obtaining smaller scars, it was not possible to prevent the development of dehiscence or generation of wide and hypertrophic scars. These results can be explained by the persistent orientation of part or whole of the wound perpendicular to the tension lines of the skin. This is reflected in the group subjected to periacetabular osteotomy by Z-shaped incisions, in which the presence of dehiscence and/or scar widening was observed only in the portions of the wound that are oriented perpendicular to the tension lines, and not in the parallel portion (Figure 8).

Considering this fact, the main author developed the oblique incision, which is oriented in parallel form to the inguinal fold and to the lines of tension in all its extension, with which wounds with edges that tend to the closing are obtained, and therefore narrower scars and without or with minimum alterations of trophism. For the same reason, we think that the probability of dehiscence is lower.

In our study group, the patients who had surgery with the oblique incision presented with narrower scars and greater aesthetic satisfaction in the survey conducted, with both results being statistically significant. In the rest of the variables analyzed, a tendency toward better results is observed for scar depression (0), hypertrophic scars (0), hyperpigmentation (2%), and dehiscence (0), but statistically significant results were not found.

It is important to remember that in the article in which the surgical technique of the oblique incision was published by the main author, no differences were observed in the surgical results of patients submitted to Bernese periacetabular osteotomies when compared with the longitudinal incision.

## Conclusion

The oblique incision presents better aesthetic results and patient satisfaction, presenting fewer complications related to the surgical wound and healing process, compared with the original, straight, shortened, and Z-type incisions.
